# Humoral immune response to COVID-19 mRNA vaccination in relation to selenium status

**DOI:** 10.1016/j.redox.2022.102242

**Published:** 2022-02-03

**Authors:** Kamil Demircan, Thilo Samson Chillon, Qian Sun, Raban Arved Heller, Georg Jochen Klingenberg, Ines Maria Hirschbil-Bremer, Petra Seemann, Joachim Diegmann, Manuel Bachmann, Arash Moghaddam, Lutz Schomburg

**Affiliations:** a Institute for Experimental Endocrinology, Charité-Universitätsmedizin Berlin, Corporate Member of Freie Universität Berlin, Humboldt-Universität zu Berlin, and Berlin Institute of Health, D-10115 Berlin, Germany; b Berlin Institute of Health (BIH), Biomedical Innovation Academy (BIA), D-10117 Berlin, Germany; c Bundeswehr Hospital Berlin, Clinic of Traumatology and Orthopaedics, D-10115 Berlin, Germany; d Department of General Practice and Health Services Research, Heidelberg University Hospital, D-69120 Heidelberg, Germany; e ATORG, Aschaffenburg Trauma and Orthopaedic Research Group, Center for Orthopaedics, Trauma Surgery and Sports Medicine, Hospital Aschaffenburg-Alzenau, D-63739 Aschaffenburg, Germany; f selenOmed GmbH, D-10965 Berlin, Germany; g Orthopedic and Trauma Surgery, Frohsinnstraße 12, D-63739 Aschaffenburg, Germany

**Keywords:** SARS-CoV-2, Vaccine, Antibody, Glutathione peroxidase, Cohort study

## Abstract

The essential trace element selenium (Se) is of central importance for human health and particularly for a regular functioning of the immune system. In the context of the current pandemic, Se deficiency in patients with COVID-19 correlated with disease severity and mortality risk. Selenium has been reported to be associated with the immune response following vaccination, but it is unknown whether this also applies to SARS-CoV-2 vaccines. In this observational study, adult health care workers (n = 126) who received two consecutive anti-SARS-CoV-2 vaccinations by BNT162b2 were followed for up to 24 weeks, with blood samples collected at the first and second dose and at three and 21 weeks after the second dose. Serum SARS-CoV-2 IgG titres, neutralising antibody potency, total Se and selenoprotein P concentrations, and glutathione peroxidase 3 activity were quantified. All three biomarkers of Se status were significantly correlated at all the time points, and participants who reported supplemental Se intake displayed higher Se concentrations. SARS-CoV-2 IgG titres and neutralising potency were highest three weeks after the second dose and decreased towards the last sampling point. The humoral immune response was not related to any of the three Se status biomarkers. Supplemental Se intake had no effect at any time point on the vaccination response as measured by serum SARS-CoV-2 IgG levels or neutralising potency. Overall, no association was found between Se status or supplemental Se intake and humoral immune response to COVID-19 mRNA vaccination.

## Highlights

• Humoral SARS-CoV-2 vaccination response in healthy adults was unrelated to baseline Se status or supplemental Se intake.
• Vaccine-induced neutralising activity correlated with SARS-CoV-2 IgG concentration, but not with Se status.
• Rise and decline of the vaccination-induced SARS-CoV-2 IgG was Se status-independent during 24 weeks after first dose.

## Graphical abstract

**Figure:**
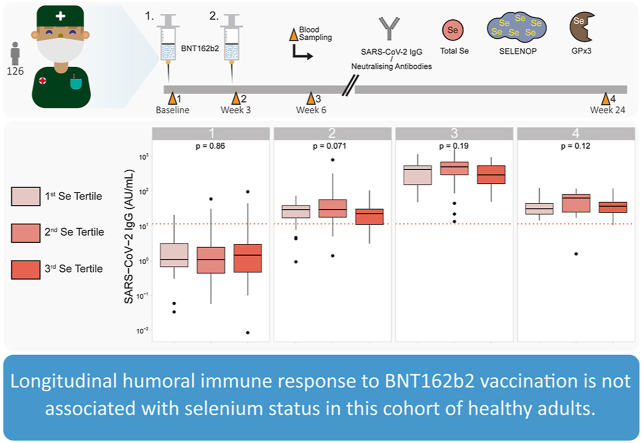

## 1 Introduction

The trace element selenium (Se) is incorporated into a set of proteins termed selenoproteins that are involved in vital functions, making this micronutrient essential for human health [1,2]. Hereby the Se status, mainly controlled by dietary intake, constitutes a limiting factor for selenoprotein expression [3,4]. A deficient Se status has been associated with several disease risks, including cardiovascular disease (CVD) [1,[5], [6], [7]] and cancer [8,9]. Further, Se is needed for many important aspects of life such as fertility [[10], [11], [12]], thyroid hormone regulation [13,14], and particularly an efficient immune system [15].

The humoral and cell-mediated immune responses critically depend on Se and selenoproteins, as shown both by experimental and clinical studies [16,17]. Se deficiency is associated with autoimmune diseases (AID) and may trigger disease onset directly by increasing ferroptosis rate of neutrophils due to insufficient expression of glutathione peroxidase 4 (GPx4) [18]. Accordingly, AID may develop in conditions of severely suppressed Se status, such as after pregnancy, severe illness or COVID19 [19,20]. A central role of Se supply is also documented in the context of viral infections. The endemic Keshan disease in areas of low habitual Se intake constitutes a most instructive example, as it develops in severely Se deficient subjects upon coxsackievirus infection [21]. Supplementation with Se proved efficient for disease prevention [22]. Similarly, Se deficiency has been associated with survival and progression of HIV-1 infection [[23], [24], [25]]. Besides other essential micronutrients like zinc, copper or vitamin D, the central role of an adequate Se status is vividly discussed in the context of the COVID-19 pandemic. Individual level [26,27], cumulative population level [28] as well as geographical data [29] have shown an inverse association of Se status and infection risk or outcome of COVID-19.

A direct association of Se status with immune response after vaccinations has been suggested [30]. A recent study described how the Se-GPx4-ferroptosis pathway regulates function of follicular T-helper cells supporting antibody production [31]. Supplementation of Se was shown to increase GPx4 expression and antibody titres following Influenza vaccination [31]. Given the wide-ranging involvement of Se for an efficient humoral and cellular immune response, we hypothesized that Se status correlates with antibody response following COVID-19 mRNA (BNT162b2) vaccination.

## 2 Material and methods

### 2.1 Study cohort

Blood sampling was conducted within the ATORG Study [32]. Ethical counselling was provided by the authorities in Bavaria, Germany (Ethik-Kommission der Bayerischen Landesärztekammer, Munich, Germany, EA No. #20033), and the study was registered at the German Clinical Trial Register (Deutsches Register Klinischer Studien, ID: DRKS00022294, Sept. 14th 2020), with an amendment of the Ethik-Kommission der Bayerischen Landesärztekammer on Jan. 12th^,^ 2021. All volunteering participants provided written informed consent for the study prior to enrolment. In brief, adult health care workers (n = 126 at baseline) received two doses of BNT162b2, Biontech/Pfizer vaccine. A serum sample was prepared at first dose, second dose (week three), week six and at week 24, shipped on dry ice to the analytical laboratory in Berlin, Germany, and analysed by scientists and technicians blinded to the clinical data, as described [26,32].

### 2.2 Assessment of SARS-CoV-2 IgG and neutralising activity

Both methods have been described in detail earlier [32]. Briefly, an automated chemiluminescent two-step capture immunoassay (TGS COVID-19, product code: CVCL100G, Immunodiagnostic Systems (ids) Holdings PLC, Frankfurt, Germany) for the automated analyser (IDS-iSYS Multi-Discipline Automated System, ids) was used to determine serum SARS-CoV-2 IgG concentrations. The neutralising activity was assessed by measuring the interference of serum samples on the binding of recombinant spike protein to SARS-CoV-2 receptor ACE2 by a competitive method (SPIA, Spike Protein Inhibition Assay, product code: DKO205/RUO, ids Holdings PLC) on the same automated analyser.

### 2.3 Quantification of total serum Se

Total serum Se concentrations were determined by total reflection X-ray fluorescence (TXRF) analysis, as described [33]. Briefly, serum samples were mixed with an internal standard containing 1000 μg/L gallium, in a 1:2 dilution, and applied to quartz glass carriers. Measurements were conducted using a TXRF spectroscope (S4 TStar, Bruker Nano GmbH, Berlin, Germany). A standard serum with validated Se concentrations (Seronorm Sero AS, Billingstad, Norway) served as control; intra- and inter-assay of coefficients of variation were below 5% during the measurements.

### 2.4 Quantification of selenoprotein P

A validated, commercial sandwich ELISA (selenOtestTM, selenOmed GmbH, Berlin, Germany) was used to assess serum SELENOP concentrations [34]. According to the manufacturer's instructions, three different control samples with low, medium and high concentrations of human SELENOP were included in all assay runs. Intra- and inter-assay coefficient of variation was below 15% during the analyses.

### 2.5 Quantification of GPx3 enzyme activity

Enzymatic activity of GPx3 was measured by a coupled enzyme activity assay [35]. Briefly, glutathione and tert-butyl-hydroperoxide served as substrates for GPx3. The decrease in NADPH during the consumption by glutathione reductase catalyzing glutathione reduction was measured at 340 nm as readout of GPx3 activity. A standard was included during all measurements and served to control the reaction conditions. Intra-assay coefficient of variation was below 12% during the analyses.

### 2.6 Statistical analysis

For all continuous variables, normality was assessed using the Shapiro-Wilk-Test. Continuous variables are presented as median with interquartile range (IQR). For the main analyses, Se biomarkers were categorized into tertiles. Comparisons between two groups were conducted applying Wilcoxon-Rank-sum test, and comparisons between three groups were conducted applying Kruskal-Wallis test. Correlation between Se biomarkers was assessed using Spearman's Rank correlation test. All analyses were two-sided and p-values below 0.05 were considered significant. The statistical analyses were performed by the R software, version 4.1.1, implementing the packages dplyr [36], tidyr [37], gtsummary [38], ggplot2 [39], ggpubr [40].

## 3 Results

### 3.1 Study cohort, vaccination and baseline SARS-CoV-2 IgG concentrations

**Fig. 1:**
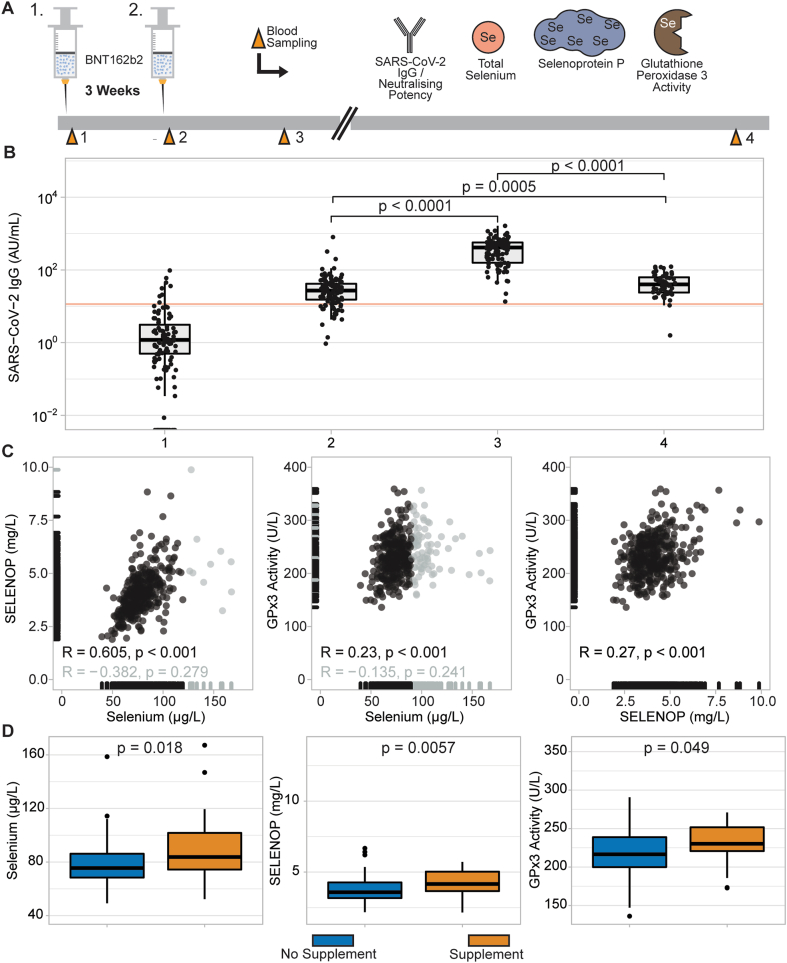
Study design, IgG response, and selenium status of the cohort. (A) Participants received two doses of BNT162b2 vaccine within a three week-interval. Blood sampling was conducted at first and second dose, three weeks and 21 weeks after the second dose. In serum samples, SARS-CoV-2 IgG titres, neutralising potency of antibodies, total Se, SELENOP, and activity of GPx3 were measured. **(B)** Most IgG titres were below the cut-off of seropositivity (11.5 AU/mL, red line) at time point of first sampling, except for eight participants. All volunteers reached seropositivity three weeks after the second vaccination. A waning of the IgG titres was observed at the last sampling time-point, however the titres were still significantly higher than baseline. A similar pattern for neutralising potency of the antibodies was observed (Fig. S1). **(C)** Serum Se and SELENOP correlated tightly below the threshold of 120 μg/L (black dots) (R = 0.605, p < 0.001), while the two parameters showed no significant correlation above 120 μg/L (gray dots). Serum Se and GPx3 activity correlated well below the threshold for GPx3 saturation (90 μg/L, black dots; R = 0.23, p < 0.001), but not above (gray dots). SELENOP and GPx3 activity correlated in the whole cohort (R = 0.27, p < 0.001). **(D)** Study participants who reported recent supplemental Se intake had higher levels of all three Se status biomarkers at baseline. Two-sided Wilcoxon-Rank-sum test was applied to detect differences between the two groups.

In this observational study, a cohort of healthy adult health care professionals received two doses of the BNT162b2 mRNA vaccine within a time interval of 3 weeks and provided serum samples for analysis. The observational period comprised the two vaccinations in addition to two later time points at six and 24 weeks after first vaccination (Fig. 1A). The majority of the participants were female (87.3% at enrolment) (TableS1). At the time of first vaccination, all but eight of the participants were IgG seronegative, with a median SARS-CoV-2 IgG titre of 0.6 AU/mL (Fig. 1B). Twenty-eight subjects reported intake of Se containing supplements.

### 3.2 Longitudinal immune response

Vaccination response was assessed by quantifying the SARS-CoV-2 IgG titres at the different time points. Already three weeks after vaccination, the majority of samples was above the threshold of positive humoral immune response, as predefined by the assay provider (indicated as horizontal orange line in Fig. 1B). A maximum was observed at sampling point three, i.e., six weeks after first vaccination, and declined concentrations were observed at 24 weeks after first vaccination (Fig. 1B). However, the IgG titres were still strongly above baseline and above the threshold for positive SARS-CoV-2 IgG titres, i.e., can be classified as seropositive. The dynamic increase and decrease of neutralising antibody titres showed a very similar trend at all the time points (Fig.S1).

### 3.3 Biomarkers of Se status during the study and according to supplement use

Three different biomarkers of Se status were quantified in all the serum samples available and correlated significantly with each other, indicating a suboptimal Se status for full expression of selenoproteins in this cohort (Fig. 1C). Established thresholds for the saturation of Se-dependent expression of SELENOP (120 μg/L) and GPx3 (90 μg/L) were used to assess the correlation of the biomarkers in more detail. As expected, total Se correlated tightly with SELENOP below the threshold (Fig. 1C, left, black dots), while no significant correlation was observed above the cut-point. Similarly, total Se was closely correlated with GPx3 activity in serum samples below the threshold (Fig. 1C, centre, black dots), but no significance was detected above the cut-point. In the full collection of samples, a significant correlation between SELENOP concentrations and GPx3 activity was observed (Fig. 1C, right). The participants reporting a supplemental Se intake at baseline had a higher Se status according to all of the biomarkers analysed (Fig. 1D).

### 3.4 Effect of Se supplementation on the humoral immune response to vaccination

**Fig. 2:**
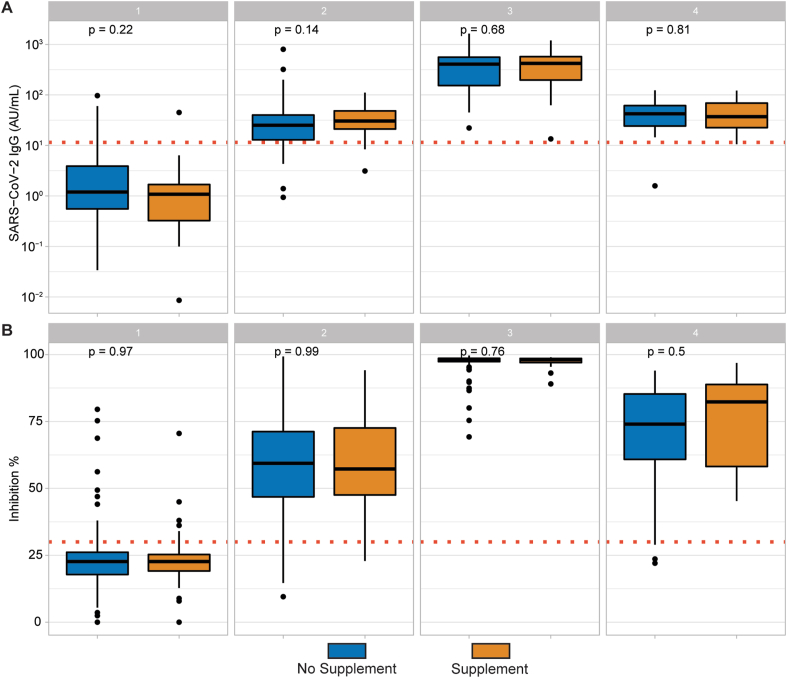
Humoral immune response according to supplementary Se intake. (A) Subjects who reported recent supplementary Se intake did not have significantly different SARS-CoV-2 IgG titres than those who did not supplement Se. **(B)** Similarly, no differences in neutralising potency (Inhibition in %) was detected between the two groups at any sampling time. Spearman's Rank test was used for correlation analysis, and two-sided Wilcoxon-Rank-sum test was applied to assess differences between groups.

The humoral immune response of the participants with self-reported Se supplementation was compared to those not reporting recent supplemental Se intake by assessing two biomarkers of immune response. Neither the SARS-CoV-2 IgG titres (Fig. 2A), nor the neutralization potency of the serum on SARS-CoV-2 spike protein binding to the ACE2 receptor (Fig. 2B) differed significantly between the two groups at any of the time points analysed.

Baseline Se status in relation to the humoral immune response to vaccination.

**Fig. 3:**
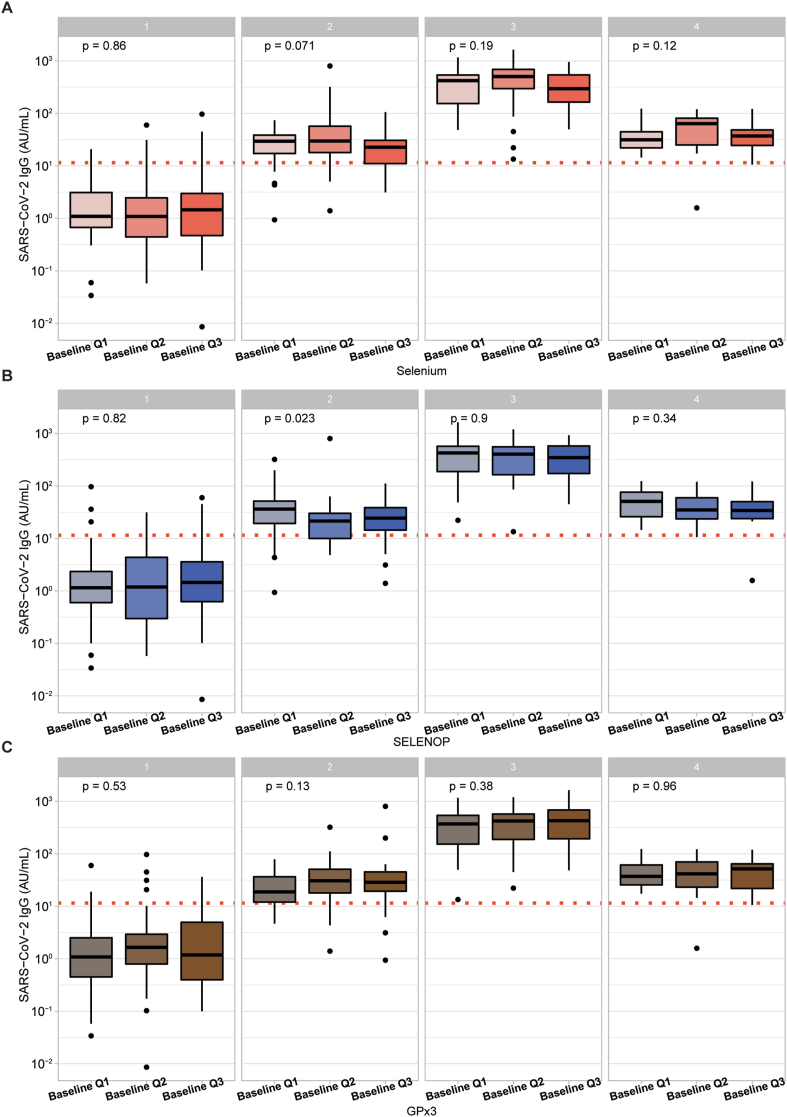
Baseline Se status in relation to SARS-COV-2 IgG titres. (A) Serum Se was categorized into tertiles (Q1; <70.8 μg/L, Q2; <82.7 μg/L, and Q3; >82.7 μg/L), and no significant differences were observed between these groups. **(B)** Serum SELENOP was divided into tertiles (Q1; <3.6 mg/L, Q2; <4.3 mg/L, and Q3; <4.3 mg/L). A significant U-shaped difference was observed at the second time point (p = 0.023). However, this observation was not retained in the follow-up. **(C)** Serum GPx3 activity was classified into tertiles (Q1; <215.3 (U/L), Q2; <248.0 (U/L) and Q3; >248.0 (U/L)), and no differences in immune response were noted across the tertiles of GPx3 activity. Two-sided Kruskal-Wallis test was used to assess differences.

Next, SARS-CoV-2 IgG titres were compared according to baseline Se status. For that purpose, all three biomarkers were categorized into tertiles (Q1-Q3) (Fig. 3). Except for a slight difference in tertiles of SELENOP, implying a U-shaped relation to IgG levels at the second sampling point (Fig. 3B), no significant differences were observed. Similarly, neutralising antibody titres were compared according to baseline Se biomarker tertiles. Again, no significant differences across the tertiles were observed (Fig.S2). As a sensitivity analysis, we compared IgG response across Se biomarker tertiles excluding participants with a reported Se intake, without identifying differences (Fig.S3). IgG titres or inhibition potency did also not differ significantly when assessing a double-deficient group in micronutrients, with a combined Se and vitamin D deficiency (Fig.S4).

## 4 Discussion

In this prospective observational study, we analysed three biomarkers of Se status in relation to time-resolved SARS-CoV-2 IgG titres and antibody neutralization potency in adult health care workers. In contrast to our hypothesis, we observed no significant association of Se status with the humoral immune response to BNT162b2 vaccine in this study. However, our cohort consisted only of healthy adults with a moderate Se status, and did not include seniors, children or adolescent subjects. Moreover, the findings should not be extrapolated to sick patients or chronically ill subjects, who may have a compromised immune system or be predisposed to severe Se deficiency.

The data are unexpected, as prior research indicated a beneficial effect of Se supplementation or high Se status for the immune response, e.g. upon influenza vaccination in three independent interventional studies [31,41,42]. However, the reported improvements were mainly related to cellular parameters, such as an increase in T cell proliferation, or to specific cytokines including IL-8 and IL-10. A recent study reported also positive effects of Se supplementation on the titres of induced antibodies to influenza vaccination in healthy adults [31]. Similar effects are reported from an intervention study with sodium selenite for the immune response to vaccination against poliovirus [16].

### 4.1 Strengths and limitations

Among the strengths of the study are the longitudinal nature and a relatively large cohort of healthy subjects being vaccinated in a highly coordinated manner within a similar environment and by the same protocol, minimizing variations in the vaccines and processing of the biosamples. Moreover, the analyses were conducted in parallel by the same methods and scientists, blinded to any clinical information, and the interaction analysed was assessed by two complementary biomarkers for immune response and by three biomarkers of Se status. Hereby, a relative comprehensive and reliable assessment of both the humoral immune response and the Se status was obtained, yielding moreover congruent results irrespective of the individual biomarkers used. Finally, the study period chosen proved as most fortunate to not only capture immediate response, but also the decline.

Among the limitations are that only humoral parameters were analysed, whereas cell-mediated vaccination response was not assessed. The study period still is somehow limited, and additional time points would have provided a more dynamic picture. Finally, the Se status of the subjects was neither on the high sufficient side, as usually observed in Se-replete populations such as US Americans or inhabitants of other Se-rich areas, nor did we include severely Se-deficient subjects that are residing in areas of marginal Se intake, such as central Asia or Africa. Collectively, in view of the observed lack of interrelationship of Se status biomarkers with SARS-CoV-2 IgG titres and neutralising activity, it appears unnecessary to consider Se supplementation for the antibody response to vaccination. Nevertheless, living with a low Se supply and developing a deficient Se status bears a number of other health risks ranging from infection and autoimmune to cardiovascular and cancer risks [2,15,20,43]; therefore, avoiding a severe Se deficit appears prudent and meaningful for staying healthy, in particular during the current pandemic.

## Contributions

Conceptualization, K.D., A.M. and L.S.; methodology, K.D., T.S.C., Q.S., G.J.K., P.S.; validation, K.D., T.S.C., R.A.H. and L.S.; formal analysis, K.D., T.S.C., and L.S.; resources, J.D., I.M.H.-B., M.B., A.M. and L.S.; data curation, K.D., T.S.C., Q.S., G.J.K, R.A.H., I.M.H.-B. and L.S.; writing—original draft preparation, K.D., and L.S.; writing—review and editing, A.M., R.A.H., I.M.H.-B., J.D. and M.B.; software, K.D and T.S.C; visualization, K.D.; supervision, A.M. and L.S.; funding acquisition, L.S. All authors have read and agreed to the published version of the manuscript.

## Ethical approval

The study was conducted according to the guidelines of the Declaration of Helsinki, and ethical counselling was provided by the authorities in Bavaria, Germany (Ethik-Kommission der Bayerischen Landesärztekammer, Munich, Germany EA No. #20033). The study was registered at the German Clinical Trial Register (Deutsches Register Klinischer Studien, ID: DRKS00022294, September 14, 2020) with an amendment approved by the Ethik-Kommission der Bayerischen Landesärztekammer, Munich, Germany, on January 12, 2021 (EA No. #20033a).

## Data and code availability

Anonymized individual data, as well as code for statistical analyses are available upon reasonable request from the corresponding author.

## Funding

The research has been supported by the 10.13039/501100001659Deutsche Forschungsgemeinschaft (10.13039/501100001659DFG), Research Unit FOR-2558 “TraceAge” (Scho 849/6–2), 10.13039/501100003383CRC/TR 296 “Local control of TH action” (LocoTact, P17), by the German 10.13039/501100006360Federal Ministry for Economic Affairs and Energy (BMWi, ZIM program, project #KK5051601BM0), and the 10.13039/501100017268BIH, 10.13039/501100017268Berlin Institute of Health, Berlin, Germany (towards the doctoral thesis of KD).

## Declaration of competing interest

L.S. and P.S. hold shares, and P.S. serves as CEO of selenOmed GmbH, a company involved in Se status assessment. There are no other activites or relationships that may have influenced the submitted work.
