# Supplementary material for: Humoral immune response to COVID-19 mRNA vaccination in relation to selenium status
Source: Redox Biol. 2022 Feb 3;50:102242. doi: 10.1016/j.redox.2022.102242 (PMC8810594; doi:10.1016/j.redox.2022.102242)
Supplement: Multimedia component 1 [file mmc1.docx]

# Supplementary Material

**Table S1. Baseline characteristics of participants.**

| Characteristic | **Overall**, N = 126 | **Female**, N = 110 | **Male**, N = 16 |
| --- | --- | --- | --- |
| **Age** (Years) | 47 (37, 55) | 47 (37, 55) | 42 (36, 53) |
| **SARS-CoV-2 IgG** (AU/mL) | 0.6 (0.0, 2.0) | 0.6 (0.0, 2.0) | 0.6 (0.0, 2.5) |
| **Neutralising Potency** (%) | 23 (18, 26) | 23 (18, 26) | 22 (14, 25) |
| **Selenium** (µg/L) | 77 (69, 87) | 76 (68, 87) | 81 (73, 91) |
| **SELENOP** (mg/L) | 3.78 (3.23, 4.32) | 3.78 (3.23, 4.31) | 3.84 (3.45, 4.38) |
| **GPx3 Activity** (U/L) | 222 (203, 240) | 220 (200, 240) | 232 (219, 258) |
| **Selenium Supplement** | *28 (22%)* | *25 (23%)* | *3 (19%)* |
| Median (IQR); *n (%)* | | | |

*
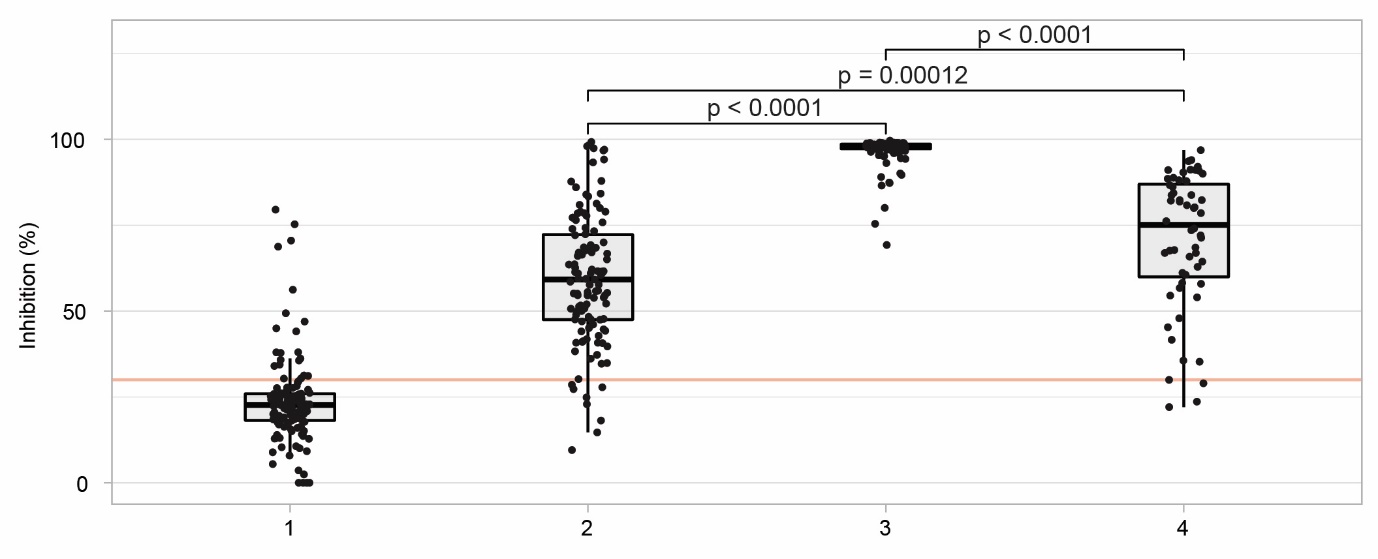
*

**Fig. S1. Longitudinal dynamics of neutralising potency of the antibodies.** The neutralizing activity of the serum samples was analysed at the different time points of first (1) and second (2) vaccination, and after additional 3 (3) and 21 (4) weeks. A characteristic and expected increase and decline in the neutralising activity was observed, correlating strongly to the concentrations of induced SARS-CoV-2 antibodies.

**
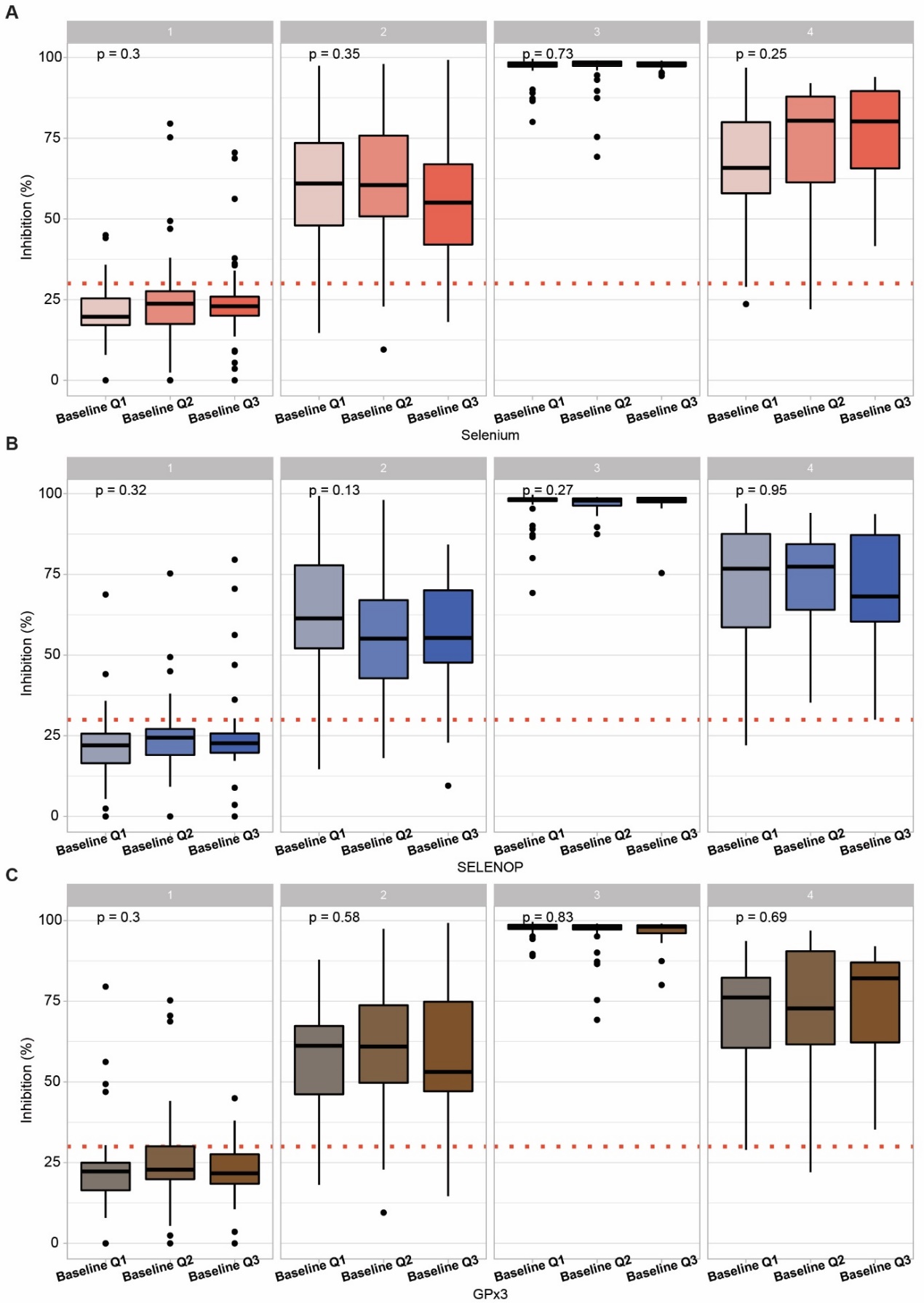
**

**Fig. S2. Baseline Se status in relation to neutralising potency of antibodies.** **(A)** Serum Se was categorized into tertiles (Q1; < 70.8 µg/L, Q2; < 82.7 µg/L, and Q3; >82.7 µg/L), but no significant differences in inhibition potencies were observed. **(B)** Serum SELENOP was divided into tertiles (Q1; < 3.6 mg/L, Q2; < 4.3 mg/L, and Q3; < 4.3 mg/L), and no significant differences in neutralising ability were detected. **(C)** Serum GPx3 activity was classified into tertiles (Q1; < 215.3 (U/L), Q2; < 248.0 (U/L) and Q3; > 248.0 (U/L)), and no differences in inhibition potency were found across the tertiles. Two-sided Kruskal-Wallis test was used to assess differences.


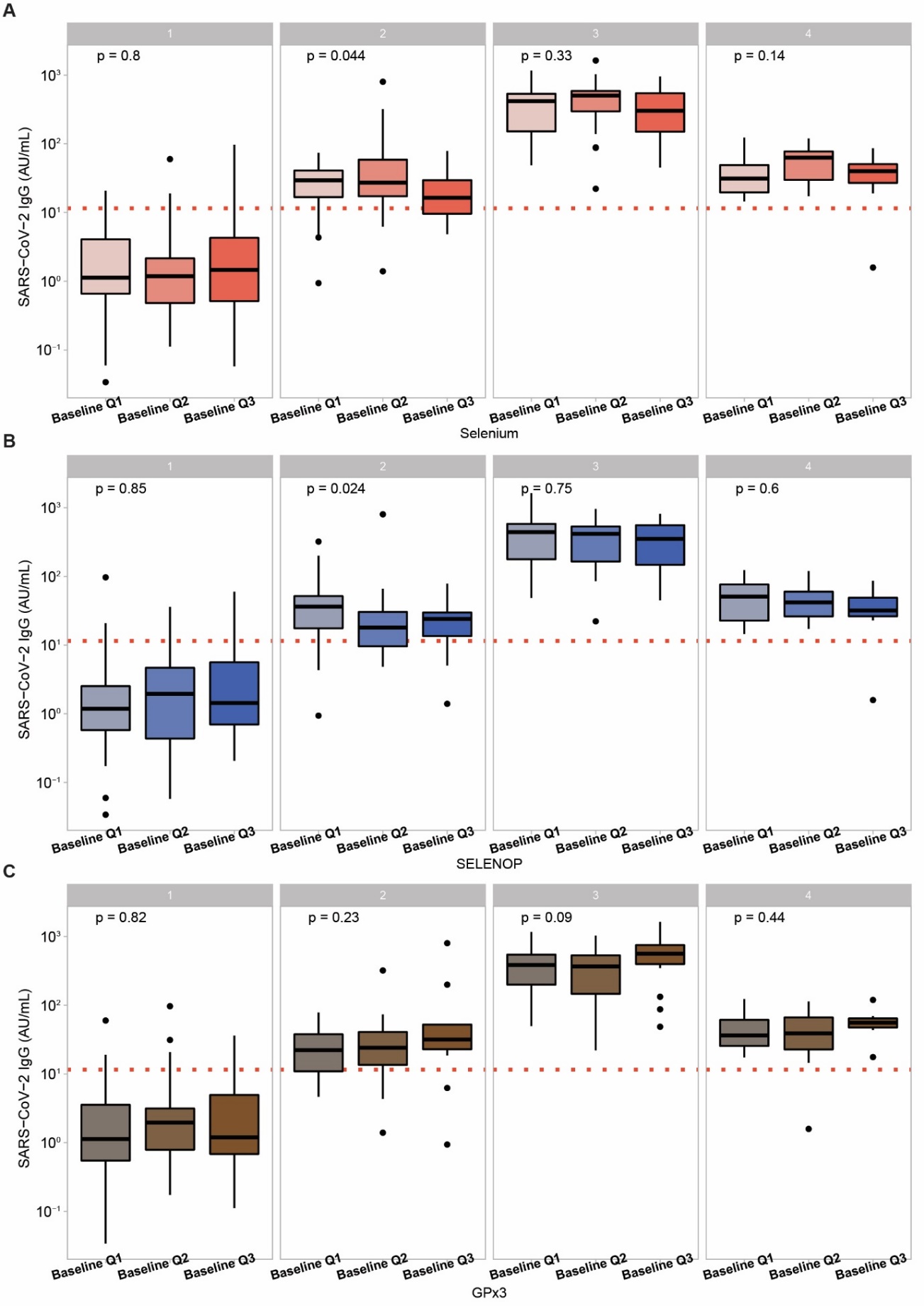


**Fig. S3. Sensitivity analysis excluding participants who reported supplementary Se intake.** IgG response was compared across tertiles of Se biomarkers at baseline excluding health care workers who have reported supplementary intake. No significant difference was observed, except for the second sampling regarding total Se and SELENOP **(A,B)**.


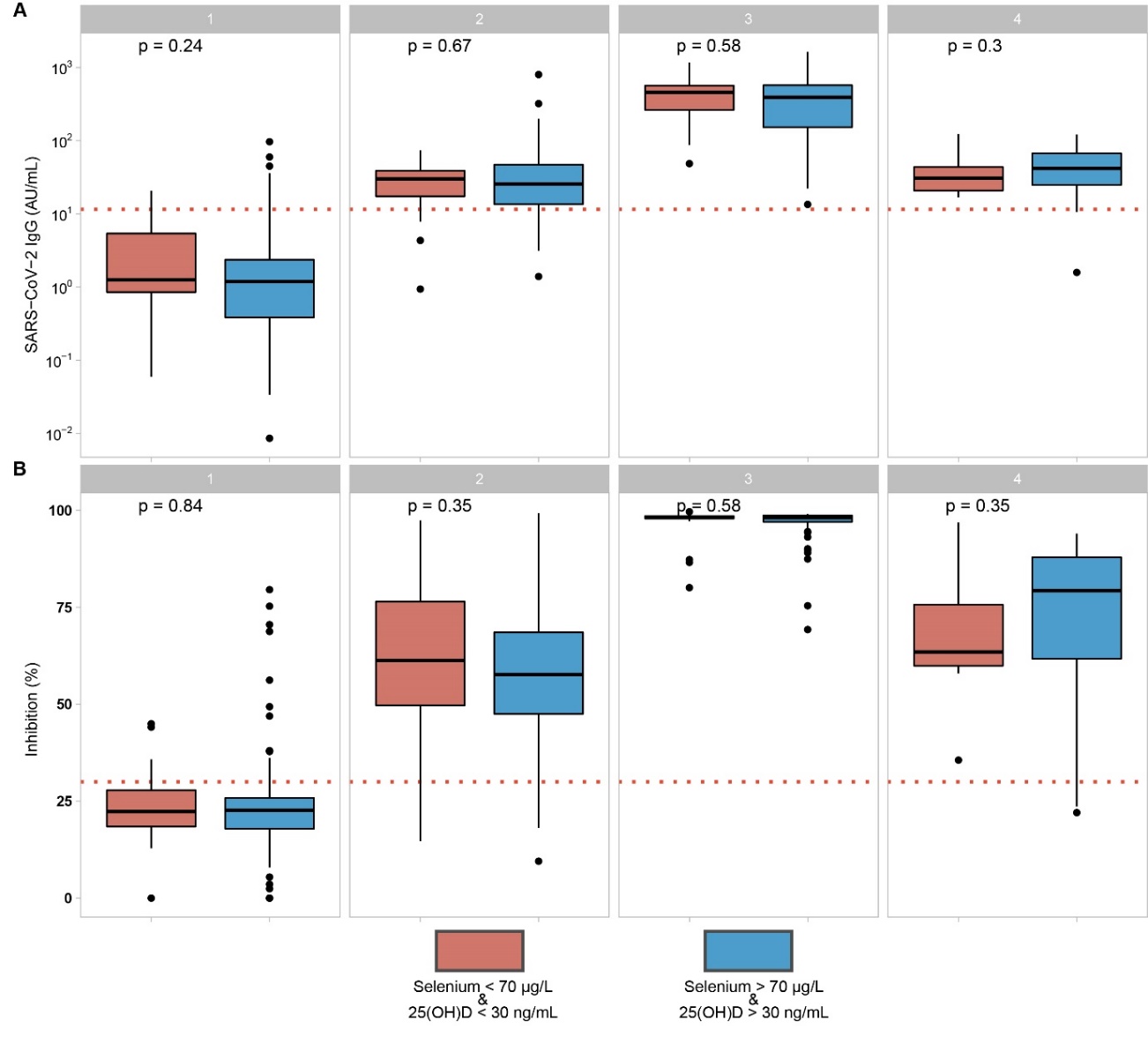


**Fig. S4. Combined Se and 25(OH)D deficiency in relation to SARS-COV-2 IgG titres.** Double deficiency was defined as Se < 70 µg/L and 25(OH)D < 30 ng/mL measured at baseline. **(A)** When comparing the IgG titres, or **(B)** the inhibition potency according to double deficiency status, no significant differences were observed. Two-sided WilcoxonRank-sum test was used to assess differences.
